# Supporting the Needs of Adolescents and Young Adults: Integrated Palliative Care and Psychiatry Clinic for Adolescents and Young Adults with Cancer

**DOI:** 10.3390/cancers13040770

**Published:** 2021-02-12

**Authors:** Mohamed Abdelaal, Pamela J. Mosher, Abha Gupta, Breffni Hannon, Christine Cameron, Malka Berman, Rahim Moineddin, Jonathan Avery, Laura Mitchell, Madeline Li, Camilla Zimmermann, Ahmed al-Awamer

**Affiliations:** 1 Department of Supportive Care, University Health Network, Toronto, ON M5G 2C4, Canada; mohamed.abdelaal@uhn.ca (M.A.); pamela.mosher@uhn.ca (P.J.M.); Breffni.Hannon@uhn.ca (B.H.); Madeline.li@uhn.ca (M.L.); camilla.zimmermann@uhn.ca (C.Z.); 2 Princess Margaret Cancer Centre, University Health Network, 620 University Ave., Toronto, ON M5G 2C1, Canada; abha.gupta@sickkids.ca (A.G.); christine.cameron@uhn.ca (C.C.); malka.berman@uhn.ca (M.B.); laura.mitchell@uhn.ca (L.M.); 3 Division of Palliative Medicine, Department of Medicine, University of Toronto, 6 Queen’s Park Crescent West, Toronto, ON M5S 3H2, Canada; 4 Division of Child and Adolescent Psychiatry, Hospital for Sick Children, 555 University Ave., Toronto, ON M5G 1X8, Canada; 5 Department of Psychiatry, University of Toronto, 1 King′s College Cir, Toronto, ON M5S 1A8, Canada; 6 Division of Medical Oncology and Haematology, Department of Medicine, University of Toronto, 6 Queen’s Park Crescent West, Toronto, ON M5S 3H2, Canada; 7 Division of Hematology/Oncology, Hospital for Sick Children, 555 University Ave., Toronto, ON M5G 1X8, Canada; 8 Department of Family and Community Medicine, University of Toronto, 500 University Ave., Toronto, ON M5G 1V7, Canada; rahim.moineddin@utoronto.ca; 9 School of Nursing, University of British Columbia, T201-2211 Wesbrook Mall, Vancouver, BC V6T 2B5, Canada; jonathan.avery@uhnresearch.ca

**Keywords:** adolescents and young adults, palliative care, symptom management, end of life, psychosocial, psychiatry

## Abstract

**Simple Summary:**

Adolescents and young adults (AYAs) with cancer experience a high level of distress and have unique unmet palliative and supportive care needs. There is limited knowledge about the symptom burden, quality of life, and type of care that AYA patients receive. In 2017, a dedicated AYA-specialized palliative care clinic was established at Princess Margaret Cancer Centre in Canada, with a collaborative approach between palliative care and psychiatry. This study aims to describe the demographics and symptoms burden of AYA cancer patients who attended the integrated palliative care and psychiatry clinic, measure the impact of the clinic on AYAs’ symptom control, and examine their end-of-life outcomes.

**Abstract:**

Clinical guidelines aimed at cancer care for adolescents and young adults (AYAs) encourage early integration of palliative care, yet there are scarce data to support these recommendations. We conducted a retrospective chart review of AYA patients, aged 15 to 39 years, who were referred to the Integrated AYA Palliative Care and Psychiatry Clinic (IAPCPC) at the Princess Margaret Cancer Centre between May 2017 and November 2019 (*n* = 69). Demographic data, symptom prevalence, change in symptom scores between baseline consultation and first follow-up, and intensity of end-of-life care were collected from the patients’ medical charts, analyzed, and reported. Of the 69 patients, 59% were female, and sarcoma was the most common cancer. A majority of patients had at least one symptom scored as moderate to severe; tiredness, pain, and sleep problems were the highest scored symptoms. More than one-third used medical cannabis to manage their symptoms. Symptom scores improved in 61% after the first clinic visit. Out of the 69 patients, 50 (72.5%) had died by October 2020, with a median time between the initial clinic referral and death of 5 months (range 1–32). Three patients (6%) received chemotherapy, and eight (16%) were admitted to an intensive care unit during the last month of life. In conclusion, AYAs with advanced cancer have a high burden of palliative and psychosocial symptoms. Creating a specialized AYA palliative care clinic integrated with psychiatry showed promising results in improving symptom scores and end-of-life planning.

## 1. Introduction

In North America, adolescents and young adults (AYAs) are defined as persons aged 15 to 39 years [1]. Cancer remains a leading cause of disease-related mortality in AYAs [2,3,4], with an increasing incidence [4,5]. Despite the improvement in AYA patients’ overall survival outcomes [5,6,7], they have unmet unique psychosocial, medical, and palliative care needs that differ from those of pediatric and older adult patients [8,9,10]. Cancer disrupts AYAs’ cognitive, psychological, and social development and impacts their identity and social life [9,10]. Most AYAs with cancer experience difficulties in achieving their personal and professional goals while facing a life-threatening disease [8]. Furthermore, this age group has a higher prevalence of psychosocial distress, and young age (<40 years) is one of the common predictors for specialized psychosocial oncology referral, regardless of the distress level [11,12]. In addition, complex pain is more prevalent in this population, which results in higher opioid dosages than for older patients with cancer [13,14]. Addressing the physical and psychosocial challenges faced by AYA patients can play a crucial role in improving the quality of AYAs’ end-of-life care [15].

It is estimated that 12–20% of AYAs die within five years of their cancer diagnosis [7,16,17]. Providing end-of-life (EOL) care for AYAs with cancer can be challenging for healthcare providers [18,19,20,21]. Palliative care providers also face personal challenges when caring for AYAs, including higher emotional distress, which impacts their overall care [18,21]. Indicators that assess the quality of EOL care and the intensity of medical care in the last month of life for AYAs with cancer have been proposed; these are based on the frequency of emergency department visits, hospital admissions, intensive care unit (ICU) admissions in the last month, and the use of intravenous (IV) chemotherapy in the last 14 days of life [22,23]. Studies have demonstrated a higher prevalence of the use of medically intensive measures in AYAs compared with older patients with cancer, suggesting poor quality of EOL care [19,24,25]. Clark and Fasciano [9] distinguish AYA patients as an underserved and vulnerable population, requiring dedicated palliative care models to address their complex needs.

Studies in adults with cancer have demonstrated improved quality of life within six months after an initial outpatient palliative care consultation [26,27]. As a result, early integration of specialized palliative care with standard oncology care has been recommended for all adults with advanced cancer [28]. Clinical guidelines aimed specifically at cancer care for AYA patients similarly encourage early palliative care integration with standard oncological care [29]. Due to the complexity and the unique needs of this population, guidelines recommend creating specialized AYA teams that include palliative care [9,29,30]. Despite these recommendations, palliative and psychiatric care are not part of standard oncological care for AYA patients in most cancer centers, and there is limited evidence to support specific care models for AYAs with advanced cancer [31,32].

Considering the unique supportive care needs of AYA patients with advanced cancer, we developed an Integrated AYA Palliative Care and Psychiatry Clinic (IAPCPC). This study aims to retrospectively assess this model of care and evaluate the impact of providing an age-appropriate integrated palliative care and psychological support for AYAs with advanced cancer.

## 2. Materials and Methods

### 2.1. Data Sources

Princess Margaret Cancer Centre (PM) is the largest cancer care center in Canada and among the top leading cancer research centers worldwide. PM is affiliated with the University of Toronto and is part of the University Health Network (UHN). The palliative care program at PM provides daily outpatient clinics, inpatient consultation services, an acute palliative care unit, as well as a 10-bed residential hospice [33].

The AYA oncology program at PM started in April 2014. The main aim of this program is to address the supportive care needs for AYA cancer patients, including fertility, sexual health, exercise, health education, coping with cancer, and survivorship [34]. Annually, 750–800 AYAs with cancer are seen at PM. Initially, the team consisted of a medical oncologist, a clinical nurse specialist, and a project manager, and it was aligned with psychosocial and rehabilitation specialists as well. Over time, this program has expanded to include other mental health resources (e.g., psychiatrist with special focus on AYA, neuropsychologist, and AYA-specific music therapy program). However, AYA with advanced cancers continue to have unmet symptoms management and mental health needs, which were not fully addressed with the existing support. AYA referrals to palliative care were still low compared to older adults and tend to occur late in the disease course, whereas the literature and international guidelines encouraged early referrals to palliative and mental health supports [26,27,29]. The three disciplines (AYA oncology, Palliative Care, and Psychiatry) identified these gaps and collaborated to build an age-specific palliative care model integrated with psychiatry to meet the complex needs of AYA with advanced cancer.

**Figure 1:**
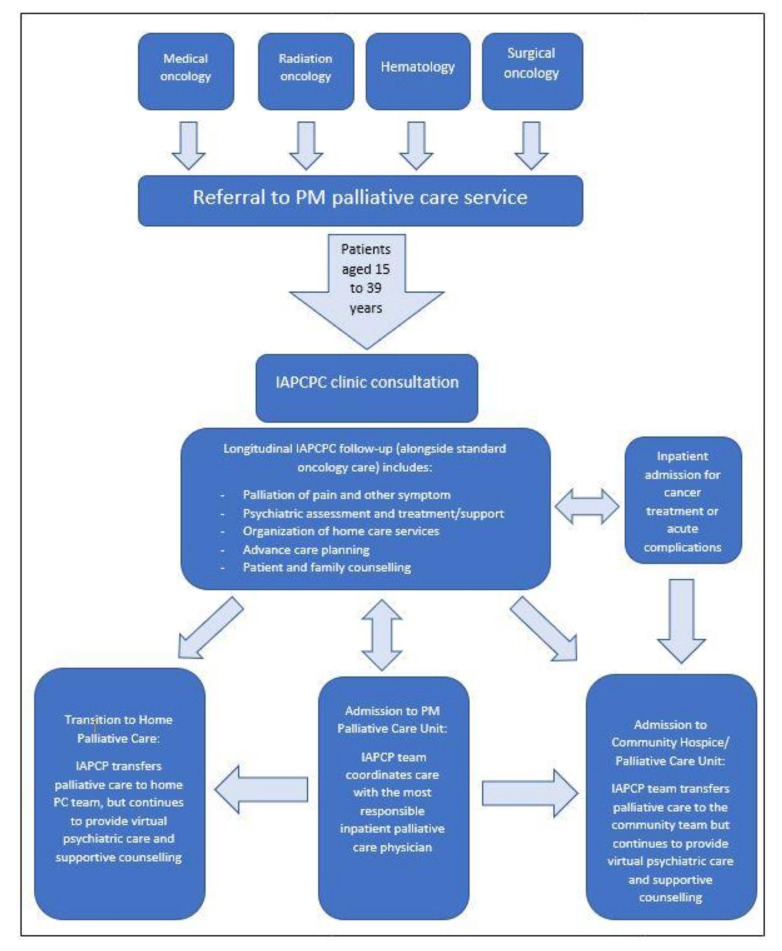
Integrated AYA Palliative Care and Psychiatry Clinic (IAPCPC) clinic model and steps of care for adolescent and young adult (AYA) cancer patients from time of referral.

The Integrated AYA Palliative Care and Psychiatry Clinic (IAPCPC) began accepting patients in May 2017. Patients are referred by their oncologists and assessed by a palliative care physician experienced in AYA, alongside an AYA psychiatrist, to optimize the care for AYA cancer patients. Figure 1 summarizes the IAPCPC clinic model and the referral process. The common reasons for referral include symptom management, advance care planning, discussions about medial cannabis, and to facilitate home care. The clinic offers care in a consultation form for AYA patients with any cancer stage, although many have metastatic disease. Although the IAPCPC accepts patients aged 15–39 years old, PM is designated as an adult center, and patients younger than 18 years are mostly treated at pediatric centers according to the Canadian healthcare system. The process of care during the initial visit is similar to that for our adult palliative care clinic [35], with a particularly strong focus on building a trusting relationship with AYAs and their families. During this first visit, the palliative care clinic nurse initially meets the patient, provides information about the palliative care services and other AYA support resources, initiates medication reconciliation, administers the modified Edmonton Symptom Assessment System scale including constipation and sleep (ESAS-r-CS) [36,37] for completion, and assesses the patient’s and family’s concerns reported on the ESAS tool. This information is reviewed with the rest of the IAPCPC team, after which the palliative care physician and the psychiatrist complete a joint consultation that lasts approximately 90 to 120 min.

During the joint assessment, the palliative care physician and psychiatrist review the patient’s cancer and treatment history, physical, psychological, spiritual and practical concerns, as well as social and home supports. The physicians use an open approach to discussion and address the physical, psychological, spiritual, and social domains, thus avoiding prestructured clinical interview questions. The goal of this format is to prioritize a fuller and more holistic understanding of each patient, to remain flexible and responsible to patient needs, and to allow AYA voices and concerns to be expressed and worked through. After discussion, the team establishes a plan of care that is reviewed with the patient and family. This plan may include pharmacological and non-pharmacological symptom management suggestions, and connection to AYA-specific support programs related to fertility, sexual health, nutrition, music therapy, and social and peer support. Non-denominational spiritual care is part of the inpatient palliative care team and accepts referrals from IAPCPC, if needed. Home care referral, when required, is also discussed. Then, the patient is provided with a follow-up plan, along with contact information for the team that includes a nursing telehealth line during business hours and out-of-hours access to an on-call palliative care physician. Follow-up is scheduled according to the patient’s preference and need. Advance care planning discussions with AYA are undertaken as an ongoing process that unfolds throughout the continuum of the disease and extends over different visits, according to the AYA’s preference and readiness. The IAPCPC team focuses on building a trusting relationship in the first few visits and addresses challenging end-of-life issues in subsequent visits after establishing a strong therapeutic relationship with AYAs and their caregivers. Earlier discussions are considered when there is an urgent need or if a patient wishes to discuss end-of-life care in the first visits.

During subsequent clinic visits, the IAPCPC team follows up on the patient’s and family’s concerns and provides necessary counseling in person or virtually. Both the palliative care physician and the psychiatrist meet with the patient and the family jointly, and then separately, to allow open communication. In between follow-up visits, AYAs and their families are encouraged to call the IAPCPC team about new or ongoing symptoms management concerns. Most of these concerns are resolved over the phone. Urgent follow up with the physicians is arranged if symptoms cannot be managed over the phone. When patients are unable to come to the clinic, they can be referred to specialized home palliative care services providing care in the community, if available. If such services are not available in the geographical region where the patient lives, telehealth appointments are provided by the IAPCPC. All patients, including those transitioned to home palliative care, continue their follow up with the IAPCPC psychiatrist either in-person or through telehealth appointments. The IAPCPC psychiatrist provides individualized counseling for AYA and their families, as needed. Additionally, PM’s social worker can also provide additional psychological counseling with the support of the IAPCPC’s psychiatrist. When appropriate, admission to the acute palliative care unit at PM is offered for complex symptom management, and the inpatient palliative care team follows up with the patient during admission [38]. Additionally, when patients are admitted to other local hospitals, the IAPCPC clinic team liaises with the hospital teams.

In addition to clinical support, the IAPCPC provides education for medical and nursing learners. Nursing and medical students, fellows, and residents from multiple disciplines, such as family medicine, internal medicine, psychiatry, and radiation oncology, follow patients at the IAPCPC with supervision. They learn about the unique needs of AYAs and about how to provide support for this population; this includes symptom management, advance care planning for AYA, communication with AYA and their families, as well as providing counseling and support for AYA and young families where psychological distress is amplified in ways that require more targeted support. In addition, the IAPCPC team provides didactic sessions, seminars, and symposia to medical learners, primary care physicians, oncologists, and the public about the AYA needs. The clinic is also an important venue for research, including prospective, retrospective, and descriptive studies.

### 2.2. Data Selection

#### 2.2.1. Study Design

This retrospective chart review was conducted for AYA patients who attended the IAPCPC clinic at PM between May 2017 and November 2019. The following information was collected from the patients’ electronic medical record: (1) demographic and oncological data; (2) clinical assessment data, including the modified Edmonton Symptom Assessment System scale including constipation and sleep ESAS-r-CS scores and Palliative Performance Status (PPS) score [39] at the time of initial consultation and at the first follow-up visit; (3) EOL measures in the last 30 days of life and death circumstances, including ICU admissions, emergency department visits, hospital admissions, chemotherapy received in the last 14 days of life, and place of death, if applicable; (4) cannabis use among AYA, as this was a common concern of AYAs during IAPCPC visits. This study was approved by the Research Ethics Board of the University Health Network. 

#### 2.2.2. Measures

The following measures are completed routinely in the IAPCPC:

ESAS-r-CS: The ESAS is a numerical rating scale that is self-rated by patients [37]. Using a scale from 0 to 10 (10 being the most severe), nine symptoms are measured (pain, tiredness, drowsiness, nausea, appetite, shortness of breath, depression, anxiety, well-being) [37]. ESAS-r is a revised version where symptoms definitions have been added [37]. The ESAS-r-CS, which has been further modified to include constipation and sleep [37], and assesses symptoms according to a time window of last 24 h, is the version used in the IAPCPC at each clinic visit. The ESAS Distress Score (EDS) is calculated by summing the nine main ESAS symptom severity scores, with prorating of scores when more than 50% of the items are completed.

PPS: The PPS is a tool that is used primarily in palliative care settings to inform decision-making [39]. It is has 5 domains—ambulation, activity level and evidence of disease, self-care, oral intake, and level of consciousness—and ranges from 0 to 100 in increments of 10, with lower scores indicating worse function [39].

### 2.3. Statistical Analysis

Descriptive statistics were calculated, including mean, median, range, and standard deviation for the continuous variables, and frequency and percentage for the categorical variables. Longitudinal analyses to describe the pattern of symptom scores change over a 6-month period were also conducted. The Wilcoxon signed-rank test was used to examine the significance of ESAS changes from baseline. All tests were two-sided, and a *p*-value less than 0.05 was considered statistically significant.

## 3. Results

### 3.1. Patients Demographics

A total of 69 AYA cancer patients were seen in the IAPCPC from May 2017 to November 2019; demographic characteristics are summarized in Table 1. The median age was 33 years (range 17–39), and 59.4% were female. Twenty-four patients (34.8%) were single, 46.4% were unemployed, and 20.3% had a history of mental illness. The most common cancer diagnosis was sarcoma (bone and soft tissue) (17.4%), followed by gynecological malignancies (13.04%), brain and central nervous system (CNS) tumors (13.04%), and gastro-intestinal (GI) malignancies (11.59%). More than 90% of the patients presented with stage IV cancer. Approximately 47.8% were cared for by their partners, whereas 26.1% listed their parents as their primary caregiver. Twenty-two patients (31.9%) reported using medical cannabis for pain and symptom management; of these 22 patients, 59.1% (13 patients) did not use any opioids.

### 3.2. Symptom Prevalence at the Initial Consultation

ESAS scores at the initial consultation are reported in Table 2. The median ESAS Distress Score (EDS) was 30 (range 5–73 out of a maximum 90). The worst symptom was tiredness (median 6/10), followed by pain and sleep (median 5 for both), and drowsiness (median 4). The majority of patients (84%) had at least one moderate-to-severe symptom rating on the ESAS score, which was rated ≥4 [40].

The mean interval between the initial consultation and the following scheduled follow-up visit was 48.5 days (SD 37.0, median 35.5 days, *n* = 50). Nineteen patients (27.5%) had only one visit to the IAPCPC, with no follow-up due to referral to home palliative care team (47.4%, *n* = 9), death (31.6%, *n* = 6) and unknown reasons (21.1%, *n* = 4). Within the first six months of IAPCPC referral, the median number of clinic visits was three (range 1–10).

### 3.3. Symptom Score Change

**Figure 2:**
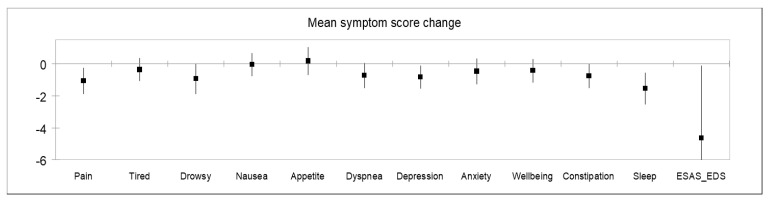
Mean symptom score change between the initial IAPCPC consultation and the first follow up.

Table 3 shows the change in symptom severity between the initial clinic consultation and the first follow-up visit (*n* = 50). Most patients reported improved or stable symptoms for constipation (87.2%), dyspnea (80.4%), depression (78.3%), sleep (76.7%), drowsiness (75.6%), anxiety (73.9%), nausea (73.9%), pain (72.9%), appetite (67.4%), tiredness (65.2%), and well-being (65.2%). The mean change in score is shown in Table 3 and Figure 2. The sleep score improved by a mean of 1.6 points (95% CI −2.6, −0.6, *p* = 0.007), pain improved by 1.1 points (95% CI −1.9, −0.2, *p* = 0.02), and depression improved by 0.8 points (95% CI −1.6, −0.1, *p* = 0.04). There was also a statistically significant improvement in EDS score by 4.7 points (95% CI −9.2, −0.1, *p* = 0.04).

### 3.4. End of Life Outcomes

By the end of October 2020, 50 patients (72.5%) had died. Table 4 describes the place of death and components of the medically intensive EOL measures for those 50 patients. The mean time between IAPCPC referral and death was 7.3 months (SD 6.6, median 5 months, range 1–32). During the last month of life, 19 patients (38%) had an emergency department visit, 24 patients (48%) were hospitalized, and eight patients (16%) had an ICU admission. Three patients (6%) received chemotherapy in the last 14 days of life. A hospice or a palliative care unit was the place of death for 18 patients (36%), followed by home (26%, *n* = 13), inpatient medical ward in a hospital (18%, *n* = 9), and ICU (12%, *n* = 6). In Canada, healthcare providers must obtain the patients’ or their substitute decision-maker consent before writing “No resuscitation” orders. Healthcare providers can not unilaterally change the resuscitation status without consent [41]. In our study, forty-four patients (88%) decided “No resuscitation” order at end of life, whereas for two patients (4%), resuscitation was attempted but unsuccessful.

## 4. Discussion

This paper describes a novel model of care to support AYAs with advanced cancer using an integrated specialized palliative care team and a psychiatrist at PM. The retrospective data describe both baseline symptom burden at the time of referral as well as the impact of the clinic on symptom severity at the first follow-up visit. Most patients had stable or improved symptom scores after only one visit to the IAPCPC clinic. We previously described findings from a qualitative study, in which patients attending the IAPCPC described benefits of age-appropriate support and improved coping with their cancer [42]. Despite some of the negative feelings reported by AYA patients toward palliative care, and initially linking IAPCPC referral with “giving up” and “losing hope” [42], this study shows that most patients seen in our clinic had stable or improved physical and psychological symptom scores after only one visit to the IAPCPC clinic. Together, these quantitative and qualitative findings suggest that this model of care is acceptable to AYAs and enables the provision of comprehensive cancer care and a reduction in suffering for this population.

### 4.1. Symptom Score Change

A large proportion of IAPCPC clinic patients initially presented with moderate-to-high symptom burden, with a median EDS score of 30 and with tiredness being the worst symptom (median 6). Similar scores have been reported for the overall palliative care outpatient population at PM [33]: our group has previously reported a median EDS score of 37 for PM patients of all ages, with tiredness consistently the highest-scored symptom (median 7). These findings are also consistent with symptom prevalence reported among AYA cancer patients in other healthcare systems. Pain and tiredness were of the most common symptoms upon referral to palliative care [43] and at end of life [44,45]. The improvement in symptom scores demonstrated in our study is encouraging for this unique model of AYA care, given that symptoms generally worsen over time in palliative care populations [46,47].

### 4.2. Late Palliative Care Referral

Given the strong evidence supporting early palliative care for patients with advanced cancer, the American Society of Clinical Oncology recommends the involvement of palliative care services in conjunction with active anti-cancer treatment for all patients with advanced cancer and high symptom burden [28]. Similarly, the National Comprehensive Cancer Network guidelines encourage the early integration of palliative care for AYA cancer patients [29]. Early palliative care involvement at least six months before death improves patients’ quality of life [26,27,48,49] and has been broadly adopted at PM. However, in our IAPCPC sample, late referrals were common. Of the 69 patients included, 21.7% had only a single IAPCPC visit, because they either died or required transition to home-based palliative care. Forty-two percent of patients died within six months of their first consultation in the IAPCPC, and the median time between AYA patients’ referral to the IAPCPC and death was 5 months.

Approximately, 750–800 new AYA cancer patients are treated at PM annually, of which 20% on average are diagnosed with cancer stages III–IV. Patients referred to IAPCPC represent only 3% of AYA cancer patients at PM and less than 15% of patients with advanced cancer. Our previous studies suggest that AYA patients and their healthcare providers resist early palliative care referral due to the stigma of palliative care and due to fear of death and losing hope [18,42]. In other healthcare systems, changing the name to “supportive care” was preferred by both oncologists and cancer patients [50,51], and it lead to earlier referrals [52].

Targeted medical education for the cancer care team and public education about AYA’s multiple needs along with discussions regarding the benefits of palliative care and psychiatry are needed to address the needs of AYAs and reduce the stigma. More research is needed to explore age-appropriate strategies to improve timely access to palliative care services for AYAs with advanced cancer, including a systemic integration of palliative care and psychiatry in AYA care.

### 4.3. End of Life Outcomes

The majority of AYAs in this study died outside of acute care settings (home or palliative care unit), with 30% (*n* = 15) dying in a hospital setting, including in the ICU. Previous studies have reported higher rates of in-hospital deaths for AYAs, ranging from 53% to 62% [19,25,44]. Patients seen in IAPCPC also had other indicators of less medically intensive EOL, compared to previous studies. In our study, 6% of AYAs received chemotherapy during the last 14 days of life, whereas other studies have reported rates as high as 40% [24,44]. Additionally, 16% of patients seen in the IAPCPC were admitted to the ICU in the last month of life, while rates as high as 22% were reported elsewhere [24]. Overall, 56% of the patients seen in the IAPCPC received at least one medically intensive EOL measure; this percentage was 68% in another study [24].

### 4.4. Medical Cannabis Use

More than one-third of AYAs attending the IAPCPC reported using medical cannabis to manage their symptoms, with 40% concurrently using opioids. These results are consistent with previous findings, with one other study reporting cannabis use among 33% of AYA cancer patients [53]. Although cannabis use has been legalized in Canada for medical and recreational purposes, its evidence base for the management of cancer-related symptoms, including pain, is sparse [54,55]. The use of medical cannabis represents another area in AYA care that warrants further research.

### 4.5. Strengths and Limitations

To our knowledge, this is the first study to describe an interdisciplinary ambulatory model for providing early palliative care for AYAs, and to report baseline and follow-up symptom scores. Our review has several limitations, including the small sample size from a single tertiary cancer center, lack of control group, and lack of data about telehealth utilization, which limit its generalizability to other settings. Our data also did not include information on referrals to social workers and other allied health due to missing data in documentation, and the fact that these referrals are available to all healthcare professionals at PM. The retrospective nature of the study, with associated missing or incomplete data, constitutes another limitation. The measures used in the IAPCPC clinic to assess the patients’ symptoms severity (ESAS-r-CS) and performance status (PPS), despite being internationally validated among the general cancer population, may not have been validated in patients less than 18 years old.

## 5. Conclusions

AYA cancer patients represent a unique group with distinctive needs, but specialized AYA clinics are uncommon, even in tertiary comprehensive cancer centers. A collaborative approach between palliative care and psychiatry at our center has demonstrated promising results in improving symptom burden and the aggressiveness of end-of-life care for this population. Further research is needed to explore the palliative care needs of this unique group, identify the most effective ways to promote early AYA referrals to palliative care from oncology teams, understand what elements of the IAPCPC model are most beneficial to patients and their caregivers as well as to healthcare providers, explore areas for improvement of the current model, and evaluate the clinic’s impact on the health care system at our cancer center. These findings will be instrumental in creating specialized AYA medical training programs and developing clinical practice recommendations.

## Figures and Tables

**Table 1:** Patient demographics (*n* = 69).

Patient Characteristics	
Age, years	
Mean ± SD *	31.52 ± 6.30
Median (range)	33 (17–39)
Patients < 18 years *n* (%)	1 (1.45%)
Gender *n* (%)	
F	41 (59.42%)
M	28 (40.58%)
Social status *n* (%)	
Single	24 (34.78%)
In a relationship	45 (65.22%)
Employment status *n* (%)	
Employed	30 (43.48%)
Unemployed	32 (46.38%)
Student	7 (10.14%)
Cancer type *n* (%)	
Brain and CNS	9 (13.04%)
Breast	4 (5.80%)
Genito-urinary (GU)	4 (5.80%)
Gastro-intestinal (GI)	8 (11.59%)
Gynecological	9 (13.04%)
Sarcoma (bone and soft tissue)	12 (17.39%)
Lung	4 (5.80%)
Head and neck	3 (4.35%)
Leukemia	5 (7.25%)
Lymphoma	1 (1.45%)
Others	10 (14.49%)
Staging at diagnosis *n* (%)	
I–III	5 (7.25%)
IV	64 (92.75%)
History of mental illness *n* (%)	
Yes	14 (20.29%)
No	55 (79.71%)
Primary caregiver *n* (%)	
Partner	33 (47.83%)
Parent	18 (26.09%)
Sibling	1 (1.45%)
Other family member	3 (4.35%)
More than one caregiver	11 (38.84%)
None	1 (1.45%)
PPS ** at baseline *n* (%)	
80–100	13 (26.00%)
60–70	30 (60.00%)
40–50	7 (14.00%)
10–30	0 (0%)
Missing	19
Medical cannabis use *n* (%)	
Yes	22 (31.88%)
No	47 (68.12%)

* SD, standard deviation; ** PPS, Palliative Performance Scale.

**Table 2:** Edmonton Symptom Assessment System scale (ESAS) scores at initial IAPCPC consultation (*n* = 69).

Variable	*N*	Mean (SD)	Median (Range)	Number of Patients Rating ≥4 (%)
Pain	68	4.6 (2.8)	5 (0–10)	19 (39.6%)
Tiredness	65	5.0 (2.8)	6 (0–10)	29 (63.0%)
Drowsiness	64	4.0 (2.9)	4 (0–9)	17 (37.8%)
Nausea	66	2.0 (2.7)	1 (0–9)	10 (21.7%)
Appetite	65	3.2 (2.7)	3 (0–10)	22 (47.8%)
Dyspnea	65	2.3 (2.6)	1 (0–10)	9 (19.6%)
Depression	64	3.2 (3.2)	2.5 (0–10)	15 (32.6%)
Anxiety	64	3.3 (3.0)	3 (0–10)	18 (39.1%)
Well-being	65	4.4 (2.6)	5 (0–9)	23 (50.0%)
Constipation	63	2.6 (2.9)	2 (0–9)	9 (23.1%)
Sleep	63	4.7 (3.0)	5 (0–10)	17 (39.5%)
ESAS_EDS	65	31.6 (17.3)	30 (5–73)	

**Table 3:** Change in symptom severity between the initial IAPCPC consultation and the first follow-up.

Variable	N *	Improve	Stable	Deteriorate	Mean Change (SD)	*p* Value **
Pain	48	27 (56.3%)	8 (16.7%)	13 (27.1%)	−1.1 (2.9)	0.02
Tiredness	46	21 (45.7%)	9 (19.6%)	16 (34.8%)	−0.4 (2.5)	0.58
Drowsiness	45	25 (55.6%)	9 (20.0%)	11 (24.4%)	−0.9 (3.3)	0.07
Nausea	46	11 (23.9%)	23 (50.0%)	12 (26.1%)	−0.04 (2.6)	0.71
Appetite	46	19 (41.3%)	12 (26.1%)	15 (32.6%)	0.2 (3.0)	0.87
Dyspnea	46	19 (41.3%)	18 (39.1%)	9 (19.6%)	−0.7 (2.8)	0.04
Depression	46	18 (39.1%)	18 (39.1%)	10 (21.7%)	−0.8 (2.5)	0.04
Anxiety	46	19 (41.3%)	15 (32.6%)	12 (26.1%)	−0.5 (2.8)	0.23
Wellbeing	46	22 (47.8%)	8 (17.4%)	16 (34.8%)	−0.4 (2.6)	0.04
Constipation	39	18 (46.2%)	16 (41.0%)	5 (12.8%)	−0.8 (2.4)	0.007
Sleep	43	24 (55.8%)	9 (20.9%)	10 (23.3%)	−1.6 (3.3)	0.26
ESAS_EDS	46	28 (60.9%)	2 (4.3%)	16 (34.8%)	−4.7 (15.8)	0.04

* Due to missing values, total number does not equal 50; ** *p* Value less than 0.05 was considered statistically significant.

**Table 4:** End-of-life care indicators in AYAs.

End-of-Life Care Indicators	*n* (%)
Chemotherapy given	
Yes	3 (6.00%)
No	45 (90.00%)
Unknown	2 (4.00%)
Emergency room visit	
Yes	19 (38.00%)
No	28 (56.00%)
Unknown	3 (6.00%)
Hospitalization	
Yes	24 (48.00%)
No	22 (44.00%)
Unknown	4 (8.00%)
ICU admission	
Yes	8 (16.00%)
No	38 (76.00%)
Unknown	4 (8.00%)
Code status at time of death	
Full resuscitation	2 (4.00%)
No resuscitation	44 (88.00%)
Unknown	4 (8.00%)
Any of above	
Yes	28 (56.00%)
No	20 (40.00%)
Unknown	2 (4.00%)
Place of death	
Home	13 (26.00%)
Hospital inpatient ward	9 (18.00%)
Intensive care unit (ICU)	6 (12.00%)
Hospice or palliative care unit (PCU)	18 (36.00%)
Unknown	4 (8.00%)

## Data Availability

The data presented in this study are available on request from the corresponding author. The data are not publicly available due to privacy restrictions and patients’ confidentiality.
